# Supplementary material for: COVID-19 Vaccination Effectiveness in the General Population of an Italian Province: Two Years of Follow-Up
Source: Vaccines (Basel). 2023 Aug 4;11(8):1325. doi: 10.3390/vaccines11081325 (PMC10459219; doi:10.3390/vaccines11081325)
Supplement: Supplementary file 1 [file vaccines-11-01325-s001.zip › vaccines-2525127-supplementary.pdf]

Table S1. Outcomes of the study among a subset of individuals with no hypertension, COPD, CVD, kidney disease, diabetes, and cancer, and no hospital admissions for any cause during the triennium 2018-2020.

	Unvaccinated	1 Dose [A]	2 Doses [B]	3/4 Doses [C]	P-value [E]	Total Sample
SARS-CoV-2 infection						
Overall sample [D], n	39,386	4894	35,806	110,263		190,349
Positive swabs, % (n)	33.9 (13,360)	32.2 (1577)	60.4 (21,629)	34.8 (38,397)	V	39.4 (74,963)
HR (95% CI) *	1 (ref. cat.)	1.37 (1.30-1.44)	2.90 (2.83-2.96)	1.50 (1.47-1.54)		--
COVID-19						
Overall sample [D], n	20,283	2180	30,219	64,877		117,559
% (n)	7.96 (1615)	3.07 (67)	1.59 (481)	1.64 (1063)		2.74 (3226)
HR (95% CI) *	1 (ref. cat.)	0.23 (0.12-0.43)	0.11 (0.08-0.14)	0.06 (0.05-0.07)		--
COVID-19 related deaths						
Overall sample [D], n	13,166	7292	27,472	69,629		117,559
% (n)	4.38 (577)	0.88 (64)	0.84 (232)	0.93 (650)		1.30 (1523)
HR (95% CI) *	1 (ref. cat.)	0.22 (0.08-0.61)	0.33 (0.20-0.52)	0.14 (0.10-0.20)		--
All-cause deaths						
Overall sample [D], n	44,989	11,452	47,610	186,676		290,727
% (n)	4.41 (1986)	2.84 (325)	4.13 (1964)	1.36 (2546)		2.35 (6821)
HR (95% CI) *	1 (ref. cat.)	1.53 (1.10-2.13)	1.91 (1.59-2.30)	0.31 (0.27-0.37)		--

HR = Hazards ratios; CI = Confidence Interval.

* Cox proportional hazards models adjusting for age and gender and infection status (all-cause death only).

[A] Please see Table 1 footnote A. [B] Please see Table 1 footnote B. [C] Please see Table 1 footnote C. [D] The number of subjects in each vaccine group varied according to the outcome, depending on the date in which the outcome occurred (before or after each vaccine dose). As an example, if a subject who received three vaccine doses had a positive SARS-CoV-2 swab after the second dose, but before the third dose, he/she was included in the group "two doses only" for the analyses of the outcome "infection", and in the group "three or four doses" for the outcome "death". As the outcome "death" included all vaccine doses (as all were administered before), the numbers in the Table are referred to the analyses of this outcome. [E] Chi-squared test for the comparisons between: (I) unvaccinated vs. 1 dose group; (II) unvaccinated vs. 2 doses group; (III) unvaccinated vs. ≥3 doses group; (IV) 1 dose vs. 2 doses group; (V) 1 dose vs. ≥3 doses group; (VI) 2 doses vs. ≥3 doses group. When not reported, p-values were <0.05.

Table S2. Outcomes of the study among the subjects with hypertension, diabetes or cancer.

	Unvaccinated	1 Dose A	2 Doses B	3/4 Doses C
Subjects with hypertension	HR (95% CI) *	HR (95% CI) *	HR (95% CI) *	HR (95% CI) *
SARS-CoV-2 infection	1 (ref. cat.)	1.08 (0.92-1.28)	1.19 (1.11-1.27)	0.69 (0.66-0.73)
COVID-19	1 (ref. cat.)	0.54 (0.39-0.75)	0.34 (0.30-0.39)	0.13 (0.12-0.15)
COVID-19 related deaths	1 (ref. cat.)	0.37 (0.27-0.52)	0.36 (0.30-0.44)	0.14 (0.12-0.16)
All-cause deaths	1 (ref. cat.)	1.42 (1.22-1.67)	1.24 (1.14-1.35)	0.19 (0.17-0.20)
Subjects with diabetes	HR (95% CI) *	HR (95% CI) *	HR (95% CI) *	HR (95% CI) *
SARS-CoV-2 infection	1 (ref. cat.)	0.94 (0.72-1.23)	1.08 (0.98-1.19)	0.62 (0.57-0.67)
COVID-19	1 (ref. cat.)	0.92 (0.59-1.42)	0.37 (0.30-0.45)	0.14 (0.12-0.16)
COVID-19 related deaths	1 (ref. cat.)	0.41 (0.26-0.64)	0.29 (0.21-0.40)	0.13 (0.10-0.16)
All-cause deaths	1 (ref. cat.)	1.48 (1.18-1.87)	1.12 (0.98-1.27)	0.18 (0.16-0.21)
Subjects with cancer	HR (95% CI) *	HR (95% CI) *	HR (95% CI) *	HR (95% CI) *
SARS-CoV-2 infection	1 (ref. cat.)	1.24 (0.98-1.59)	1.44 (1.30-1.58)	0.87 (0.81-0.94)
COVID-19	1 (ref. cat.)	0.75 (0.44-1.27)	0.39 (0.31-0.50)	0.19 (0.15-0.22)
COVID-19 related deaths	1 (ref. cat.)	0.40 (0.24-0.67)	0.43 (0.32-0.58)	0.19 (0.15-0.25)
All-cause deaths	1 (ref. cat.)	1.47 (1.18-1.83)	1.21 (1.07-1.36)	0.18 (0.16-0.20)

HR = Hazards ratios; CI = Confidence Interval.

* Cox proportional hazards models adjusting for age, gender, infection status (all-cause death only), and of the all other recorded comorbidities.

[A] Please see Table 1 footnote A. [B] Please see Table 1 footnote B. [C] Please see Table 1 footnote C.

Table S3. Outcomes of the study during the Omicron predominance (from 1 January, 2022 to the end of follow-up) *.

	**Unvaccinated**	**1 Dose [A]**	**2 Doses [B]**	**3/4 Doses [C]**	**P-value [E]**	**Total Sample**
SARS-CoV-2 infection						
Overall sample [D], n	40,610	5631	44,403	181,523		272,167
Positive swabs, % (n)	24.4 (9924)	30.0 (1689)	57.7 (25,614)	35.6 (64,578)		37.4 (101,805)
HR (95% CI) **	1 (ref. cat.)	1.33 (1.26-1.40)	3.41 (3.33-3.49)	1.51 (1.48-1.54)		--
COVID-19						
Overall sample [D], n	9924	1689	25,613	64,579		101,805
% (n)	2.91 (289)	2.25 (38)	1.40 (358)	1.63 (1053)		1.71 (1738)
HR (95% CI) **	1 (ref. cat.)	0.76 (0.54-1.07)	0.63 (0.54-0.74)	0.26 (0.23-0.30)		--
COVID-19 related deaths						
Overall sample [D], n	9579	1759	22,638	67,829		101,805
% (n)	1.29 (124)	1.42 (25)	0.76 (172)	0.93 (633)	I	0.94 (954)
HR (95% CI) **	1 (ref. cat.)	1.27 (0.83-1.96)	1.02 (0.81-1.29)	0.35 (0.29-0.42)		--
All-cause deaths						
Overall sample [D], n	40,265	5701	41,427	184,774		272,167
% (n)	0.98 (396)	1.19 (68)	1.34 (555)	1.31 (2427)	I, IV, V, VI	1.27 (3446)
HR (95% CI) **	1 (ref. cat.)	1.89 (1.46-2.45)	2.15 (1.89-2.45)	0.70 (0.62-0.78)		--

HR = Hazards ratios; CI = Confidence Interval.
* The 18,560 subjects who died or had a SARS-CoV-2 infection before January 1, 2022 were excluded from the sample.
** Cox proportional hazards models adjusting for age, gender, infection status (all-cause death only), diabetes, hypertension, major cardiovascular diseases, chronic obstructive pulmonary disease, kidney disease, and cancer.
[A] Please see Table 1 footnote A. [B] Please see Table 1 footnote B. [C] Please see Table 1 footnote C. [D] The number of subjects in each vaccine group varied according to the outcome, depending on the date in which the outcome occurred (before or after each vaccine dose). As an example, if a subject who received three vaccine doses had a positive SARS-CoV-2 swab after the second dose, but before the third dose, he/she was included in the group "two doses only" for the analyses of the outcome "infection", and in the group "three or four doses" for the outcome "death". As the outcome "death" included all vaccine doses (as all were administered before), the numbers in the Table are referred to the analyses of this outcome. [E] Chi-squared test for the comparisons between: (I) unvaccinated vs. 1 dose group; (II) unvaccinated vs. 2 doses group; (III) unvaccinated vs. ≥3 doses group; (IV) 1 dose vs. 2 doses group; (V) 1 dose vs. ≥3 doses group; (VI) 2 doses vs. ≥3 doses group. When not reported, p-values were <0.05.

Table S4. Outcomes of the study by most frequently administered vaccine types.

	**BNT162b2**	**mRNA-1273**	**Mixed vaccines** [A]
1 Dose only [B]	HR (95% CI) *	HR (95% CI) *	HR (95% CI) *
SARS-CoV-2 infection	1 (Ref. cat.)	0.81 (0.74-0.88)	--
COVID-19	1 (Ref. cat.)	0.62 (0.32-1.20)	--
COVID-19 related deaths	1 (Ref. cat.)	0.46 (0.26-0.80)	--
All-cause deaths	1 (Ref. cat.)	0.73 (0.57-0.93)	--
2 Doses only [C]	HR (95% CI) *	HR (95% CI) *	HR (95% CI) *
SARS-CoV-2 infection	1 (Ref. cat.)	0.78 (0.76-0.81)	1.42 (1.36-1.47)
COVID-19	1 (Ref. cat.)	0.75 (0.56-1.01)	0.80 (0.60-1.08)
COVID-19 related deaths	1 (Ref. cat.)	1.20 (0.85-1.69)	0.68 (0.30-1.55)
All-cause deaths	1 (Ref. cat.)	0.91 (0.82-1.01)	0.30 (0.19-0.49)
≥3 Doses [D]	HR (95% CI) *	HR (95% CI) *	HR (95% CI) *
SARS-CoV-2 infection	1 (Ref. cat.)	0.94 (0.92-0.97)	1.02 (1.00-1.04)
COVID-19	1 (Ref. cat.)	0.92 (0.72-1.17)	0.78 (0.68-0.89)
COVID-19 related deaths	1 (Ref. cat.)	1.53 (1.18-1.99)	0.83 (0.70-0.99)
All-cause deaths	1 (Ref. cat.)	1.71 (1.51-1.94)	0.88 (0.80-0.96)

HR = Hazards ratios; CI = Confidence Interval.
* Cox proportional hazards models adjusting for age, gender, infection status (all-cause death only), diabetes, hypertension, major cardiovascular diseases, chronic obstructive pulmonary disease, kidney disease, and cancer.
[A] Subjects who received at least two different vaccines.
[B] Please see Table 1 footnote A. [C] Please see Table 1 footnote B. [D] Please see Table 1 footnote C.
